# Feather in Wharton’s Duct: A Case Report

**DOI:** 10.7759/cureus.82641

**Published:** 2025-04-20

**Authors:** Mazyad M Alenezi, Rima M Alassaf, Jayd Saud

**Affiliations:** 1 Otorhinolaryngology - Head and Neck Surgery, Ad Diriyah Hospital, Riyadh Third Health Cluster, Ministry of Health, Riyadh, SAU; 2 Otolaryngology - Head and Neck Surgery, Qassim University, Buraidah, SAU; 3 College of Medicine, Qassim University, Buraidah, SAU

**Keywords:** foreign body, obstructive sialadenitis, submandibular gland, surgical exploration, wharton’s duct

## Abstract

This report highlights a remarkable case of a 12-year-old boy with a suspected foreign body obstructing Wharton's duct. The patient came to the clinic complaining of pain and swelling in the right submandibular area, as well as a history of using a feather to floss his teeth. The CT scan failed to detect the foreign body so, based on the detailed history the patient provided, surgical exploration was performed under general anesthesia and a 2 cm feather shaft was successfully retrieved from the duct. This case underscores the limitations of imaging techniques in certain scenarios and highlights the importance of correlating clinical history to achieve an early diagnosis and surgical intervention.

## Introduction

Wharton’s duct, also known as the submandibular duct, is an essential excretory channel draining saliva from the submandibular gland into the oral cavity. It is primarily obstructed by sialolithiasis. However, foreign bodies may also lead to ductal obstruction and subsequent sialadenitis. Foreign bodies in the Wharton duct represent a rare but clinically significant phenomenon in otolaryngology as a comprehensive literature review identified only 28 cases over a 58-year period, highlighting the infrequency of such events [1]. This is due to several protective anatomical and physiological qualities, like a small calibrated punctum, a mobile distal end, and a constant flow of saliva, which act as a defense against retrograde intrusion of a foreign body into the duct [1]. Despite these protective qualities, patients have been reported continuously with foreign body intrusion in the submandibular duct by either penetrating trauma or retrograde migration from the oral cavity [1] leading to swelling, pain, and secondary infections if untreated. They primarily affect children but can occur across all age groups [2]; different foreign bodies induce different presentations [3]. They mostly present as acute or chronic obstructive sialadenitis, most commonly due to calculi, strictures, kinks of the ductal system, mucous plugs, and rarely foreign bodies [1]. In this case report, the patient presents to the clinic with swelling and pain in his right submandibular area and a history of flossing with a feather that broke inside his mouth.

## Case presentation

**Figure 1:**
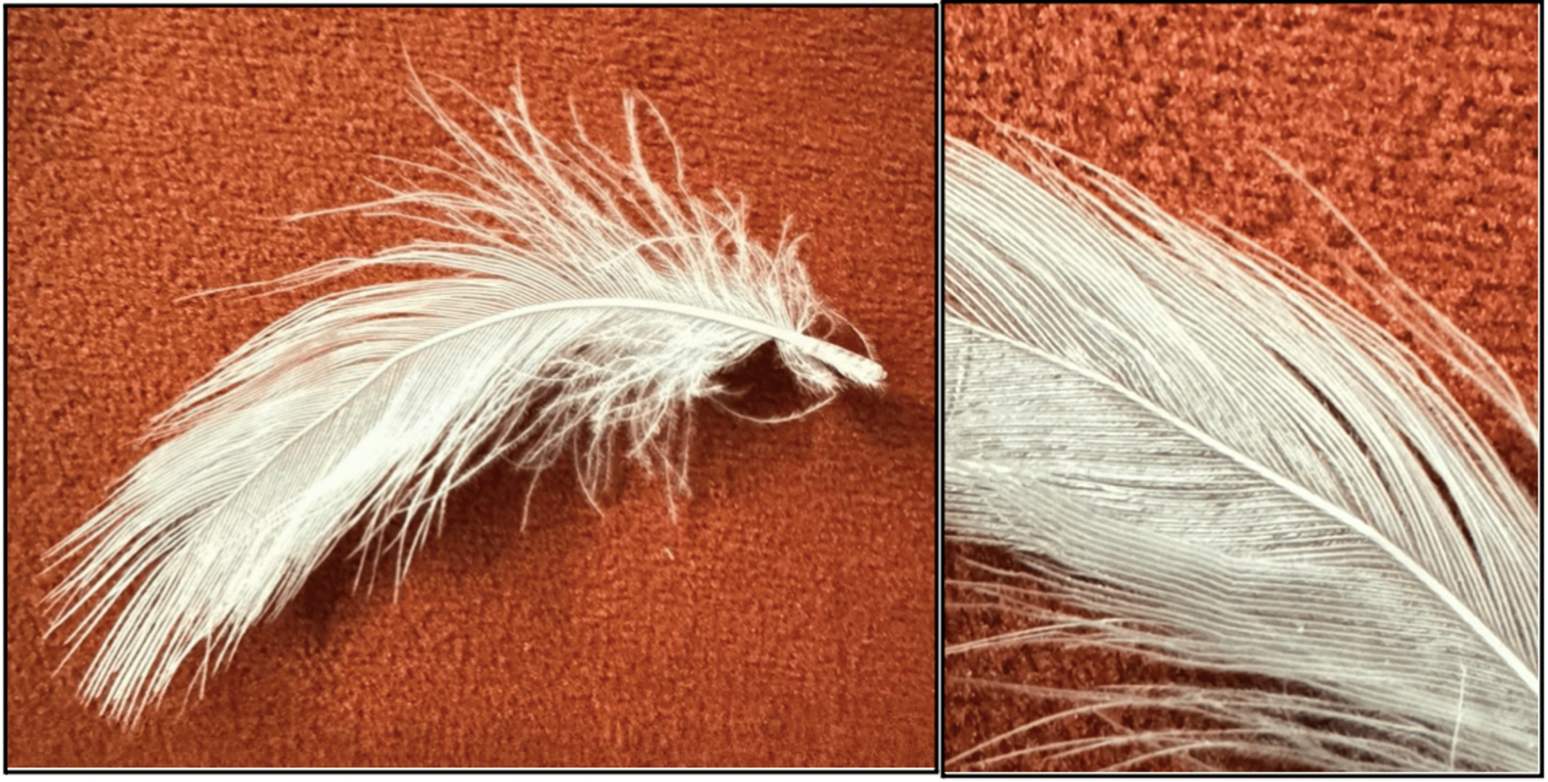
The feather used for flossing, which later caused an obstruction in Wharton’s duct.

**Figure 2:**
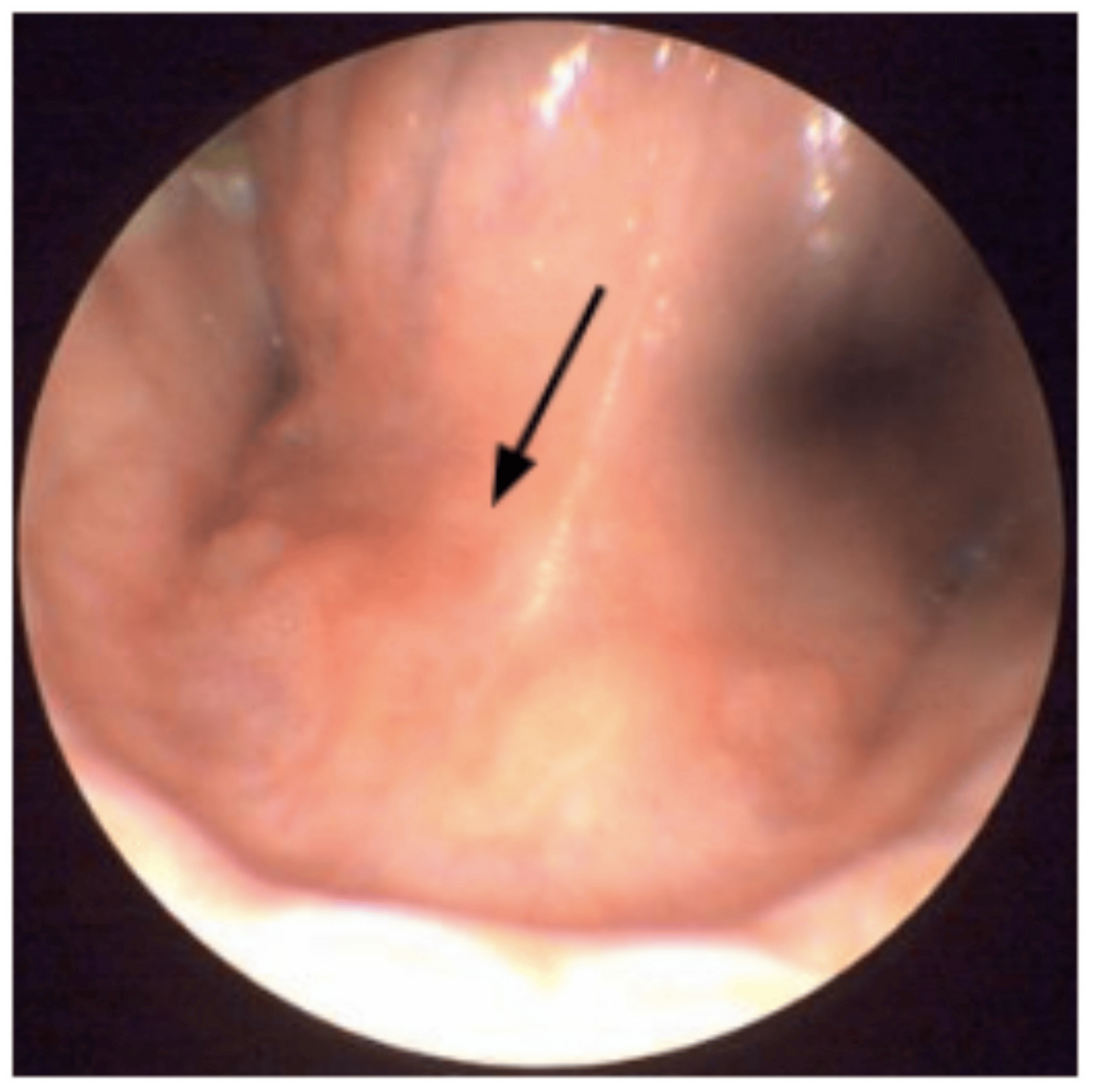
Preoperative erythema and edema observed on the right submandibular duct, indicating inflammation and obstruction.

**Figure 3:**
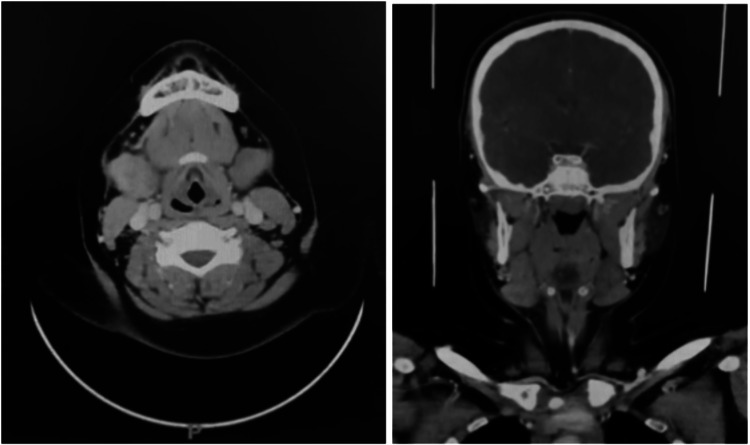
Coronal and axial view of CT scan with contrast show asymmetrical enlargement of the right submandibular gland and no foreign body was identified.

A 12-year-old male presented to the otolaryngology clinic with pain during food chewing and painful swelling in the right submandibular region for two days. The patient reported a history of using a feather to floss his teeth 10 days prior to the onset of symptoms (Figure 1). During the flossing attempt, the shaft of the feather slipped inside the right-side Wharton duct in his floor of mouth, and despite multiple attempts to retrieve it manually by eyebrow tweezers, all failed. On physical examination, erythema and edema of the right Wharton's duct were noted, along with swelling and tenderness in the right submandibular area (Figure 2). No discharge was observed on bimanual examination. A contrast-enhanced CT scan was performed, revealing asymmetrical enlargement of the right submandibular gland with faint minimal surrounding fat stranding. No evidence of abscess formation, radiopaque ductal obstruction, or glandular lesion was identified on the scan (Figure 3).

Intervention

**Figure 4:**
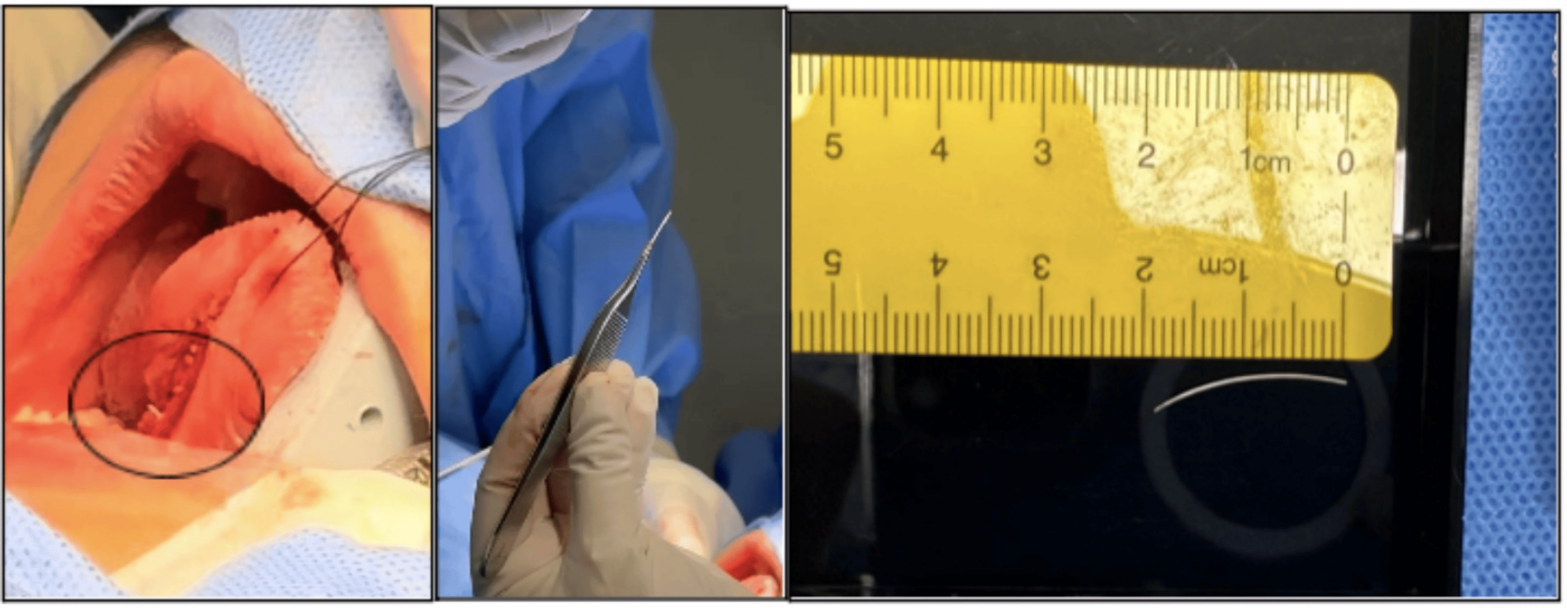
Intraoperative image showing the retrieved 2 cm-long feather shaft

**Figure 5:**
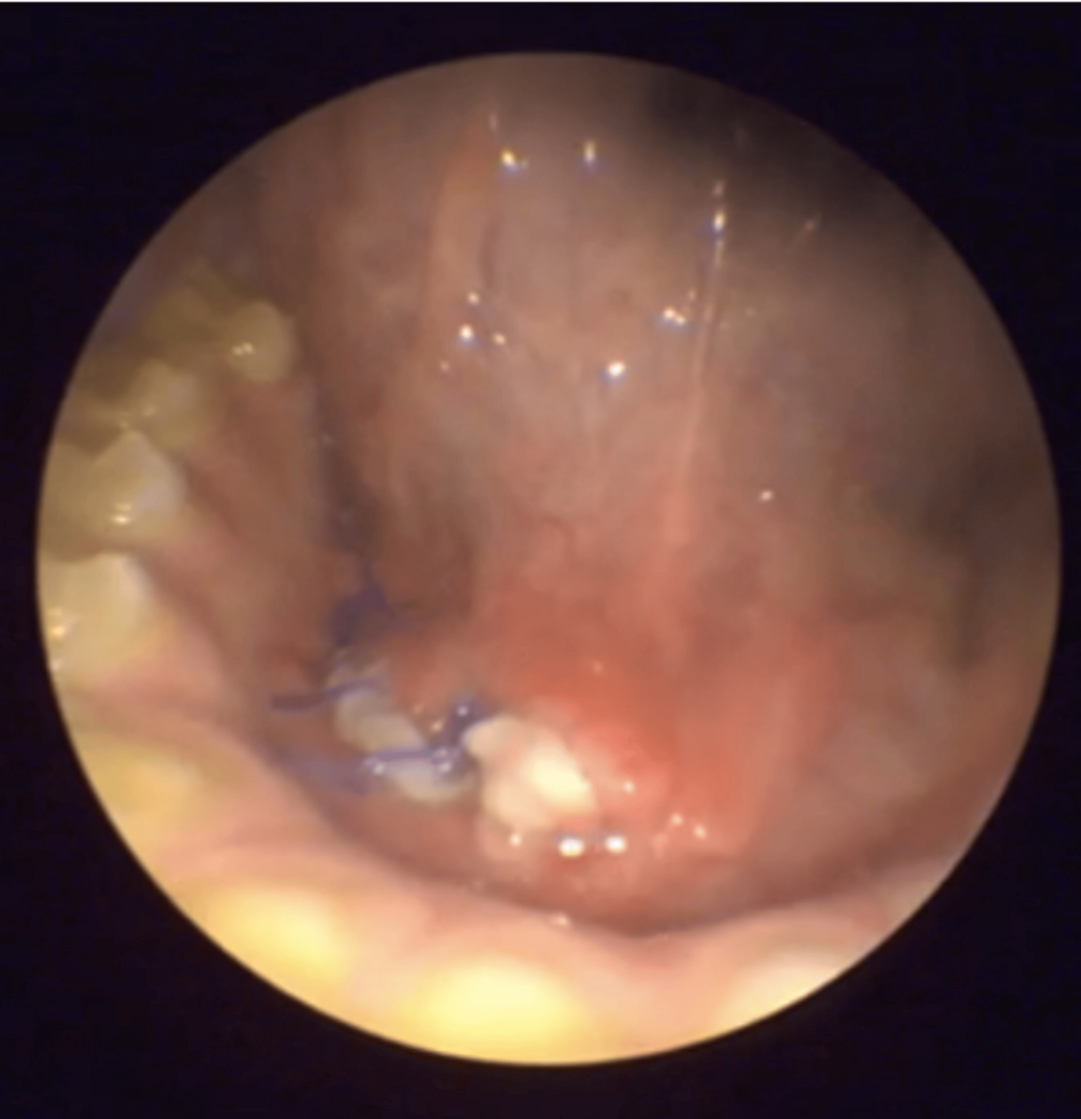
Postoperative wound healing showing no signs of infection or complications at the site of incision.

Although radiological imaging was inconclusive, the decision for exploratory surgery was made based on the patient's strong history and clinical examination, which supported the diagnosis. Our initial management focused on treating the active infection; therefore, a two-week antibiotic regimen was initiated to control the infection, reduce inflammation, and decrease local edema. Exploratory surgery was scheduled after discussing the treatment plan with the patient and his family. The procedure was performed under general anesthesia. The procedure began with an injection in the floor of the mouth with 1% lidocaine with adrenaline. A 3.0 silk was used to anchor the proximal part of Wharton's duct to minimize feather migration to the submandibular gland. Then, an incision was made parallel to Wharton's duct with Bovie cautery. After identifying Wharton's duct, an incision was made over it. A 2 cm-long feather shaft was retrieved from the right Wharton's duct. Finally, Wharton’s duct and floor of mouth mucosa were marsupialized and closed primarily with 4.0 interrupted Vicryl sutures (Figure 4). The patient had an uneventful postoperative recovery and was discharged on the same day with oral antibiotics, analgesics, and follow-up appointments. At the follow-up visit after one week, the wound on the floor of the mouth showed satisfactory healing, with no complications (Figure 5).

## Discussion

Although the occurrence of a foreign body in the submandibular gland or duct is not a common thing to encounter at the otolaryngology clinic, it has been constantly reported, with the earliest case report found being in 1962 [4] and one of the latest being the one we encountered with Dr. Alenezi at Qassim University Medical City while being on a summer training program. According to a literature review conducted in 2019 [1], the foreign bodies can have multiple origins: fingernail, hair, wooden splinter, and a fish bone. The left submandibular gland was involved in more cases (75%) than the right one (25%) [1]. Various imaging modalities, including ultrasonography, CT, magnetic resonance imaging (MRI), and high-resolution ultrasonography, are used for diagnosis. However, this case illustrates that radiolucent foreign bodies are still challenging to identify. Foreign bodies can enter salivary glands through two mechanisms: traumatic ingestion and retrograde migration via Wharton's or Stenson's duct. Foreign body removal typically requires sialendoscopy or surgery and may also involve excision of the salivary gland [5]. In our patient, it was retrograde migration and exploratory surgery was done.

A case report published in 2019 highlights a rare presentation of obstructive submandibular sialadenitis caused by a foreign body in Wharton’s duct. A 68-year-old male presented with a painful, recurrent swelling in the left side of the neck and floor of the mouth. Despite initial antibiotic treatment, symptoms persisted until clinical examination and manual ductal milking revealed a nail-like fishbone as the underlying cause. Prompt removal of the foreign body, combined with antibiotic therapy and supportive care, led to complete resolution without the need for surgical intervention or recurrence over six months of follow-up [6]. Another rare case of a foreign body in Wharton’s duct was reported in a 20-year-old male who developed a progressively enlarging neck swelling following a thorn injury. A plain X-ray of the neck lateral view revealed a well-defined circular radiopaque shadow. Surgical exploration revealed a sialolith with an embedded thorn, which was successfully removed, leading to complete resolution [7].

With early diagnosis and appropriate management, the prognosis for foreign body obstruction in Wharton’s duct is excellent. Several studies highlight that delays in treatment can result in complications associated with Wharton’s duct obstructions, like recurrent infections, abscess, and permanent glandular injury, underscoring the importance of prompt diagnosis and timely intervention [8]. A review from Capaccio et al.[9] stressed that obstructive sialadenitis, whether caused by calculi or foreign bodies, can lead to irreversible glandular destruction if not treated in a timely fashion. McGuirt et al.[2] noted that lack of timely action in units of foreign body salivary gland obstruction led to recurrent infections and more complex surgical interventions. Other cases in the literature, like those of Shehata et al. [3], remind us of the necessity of clinical correlation with imaging in the absence of definite findings. Another study by Bansal et al. [10] described a case of a persistent foreign body in the submandibular gland that did not cause an inflammatory response, which made diagnosis more challenging. In this case, even though there was no foreign body evident in the CT scan, the medical history collected was quite adequate and helpful for guiding surgery. Chowdhary et al. [11] also documented a case whereby the foreign body was only found intraoperatively during submandibular gland excision, and clinical examination and imaging were unremarkable at presentation. Like ours, this case emphasizes the crucial part patient history plays in guiding diagnosis and treatment and the need to keep a high index of suspicion even in cases where first investigations are inconclusive. 

Post-operative complications following surgical removal of foreign bodies include tingling at the tip of the tongue, swelling of the floor of the mouth, lingual nerve injury, ranulas, strictures, and infections that may develop during follow-up [9]. At the follow-up after eight months, our patient showed complete recovery without any documented consequences.

## Conclusions

We infrequently find foreign bodies in the Wharton's duct, and diagnosing them can be difficult, especially when traditional imaging procedures fall short. This example emphasizes the importance of surgical exploration as a definitive diagnostic and therapeutic approach in the face of ambiguous imaging results. The effective detection and removal of foreign material in this patient demonstrates the necessity of incorporating a thorough clinical history and physical examination results into management decisions. This case serves as an important example that positive outcomes can be obtained even in the face of diagnostic problems, with a comprehensive medical evaluation and immediate attention.
